# Full Quantitative Analysis of Arbitrary Cylindrically Polarized Pulses by Using Extended Stokes Parameters

**DOI:** 10.1038/srep17797

**Published:** 2015-12-10

**Authors:** Masato Suzuki, Keisaku Yamane, Kazuhiko Oka, Yasunori Toda, Ryuji Morita

**Affiliations:** 1 Department of Applied Physics, Hokkaido University, Sapporo 060-8628, Japan

## Abstract

Cylindrically polarized (CP) modes are laser beam modes which have rotational symmetry of the polarization distribution around the beam axis. Considerable attention has been paid to CP modes for their various applications. In this paper, by using the extended Stokes parameters and the degree of polarization defined for the spatial distribution (DOP-SD), we fully-quantitatively characterize the spectrally-resolved polarization states of arbitrary CP (axisymmetrically polarized and higher-order cylindrically polarized) broadband pulses generated by coherent beam combining. All the generated pulse states were fully-quantitatively analyzed for the first time and proved to have high symmetry (DOP-SD ≳ 0.95) and low spectral dependence of polarization states. Moreover, we show the DOP-SD, which cannot be defined by the conventional higher-order and hybrid Stokes parameters, enables us to make a quantitative evaluation of small degradation of rotational symmetry of polarization distribution. This quantitative characterization with high precision is significant for applications of precise material processing, quantum information processing, magneto-optical storage and nonlinear spectroscopic polarimetry.

A cylindrically polarized (CP) beam or Laguerre-Gauss (LG) vector vortex beam, a solution to the paraxial wave equation, is a laser beam mode which has rotational symmetry of the polarization distribution around the beam axis^1^. Its nonuniform polarization distribution has recently attracted much attention for applications such as telecommunications^2^,^3^,^4^, quantum information^5^,^6^,^7^, optical trapping^8^,^9^,^10^, physical properties measurement^11^,^12^, super-resolution microscopy^13^, detection of dielectric nano particles^14^, determination of orientation of point defects^15^ and laser processing^16^,^17^,^18^.

To realize these applications, many light sources and optical components generating CP beams have been developed^19^. Coherent beam combining methods^20^,^21^,^22^,^23^, being capable of generating arbitrary CP beams, are especially more versatile than other methods like direct producing from a resonator^24^,^25^,^26^,^27^ or using polarization converters^28^,^29^,^30^,^31^,^32^,^33^. To optimize the generation methods, it is crucial to establish quantitative evaluation method. For example, the purity evaluation of a CP beam is indispensable for the performance assessment of the application system using the CP beam, such as precision and reliability. However, almost all studies have made qualitative evaluation for CP beams. Chen *et al.*^23^ and D’Ambrosio *et al.*^33^ have characterized arbitrary CP beams by using the higher-order^34^,^35^ and the hybrid^36^ Stokes parameters. Not being able to define the degree of polarization^37^, these parameters cannot be responsible for the local deviation, in the symmetry of CP states, which often appears in experiments. As far as we know, all of the studies including their reports therefore did not make the fully-quantitative characterization of the generated CP beams, but qualitative or partly-quantitative characterization. In other words, the purity of CP beams generated so far has not been experimentally certified. To overcome these issues, we have proposed the extended Stokes parameters (ESPs) and their degree of polarization for the spatial distribution (DOP-SD; modified DOP)^37^,^38^, and have already shown their availability of quantitative characterization of *l* = 1 CP narrowband pulses^38^ (the pulses having *C*_∞_ symmetry of their transverse electric fields; the definition of *l* is described in our report^37^). In the present paper, to demonstrate the importance of the fully-quantitative characterization of CP beams, we generate *l* = 1 and *l* = 2 CP broadband pulses and make fully-quantitative spectrally-resolved characterization by using the ESPs and their DOP-SD. To our knowledge, the fully-quantitative characterization of various CP broadband pulse states is conducted for the first time. CP pulses recently began to be used in some applications such as material processing^39^ and nonlinear spectroscopic polarimetry^40^, where broadband or ultrashort CP pulses give us more information in the frequency or temporal domain. In this sense, the fully-quantitative characterization of broadband or ultrashort CP pulses here is significant.

## Results and Discussions

### Arbitrary manipulation of cylindrically polarized pulse states

**Figure 1:**
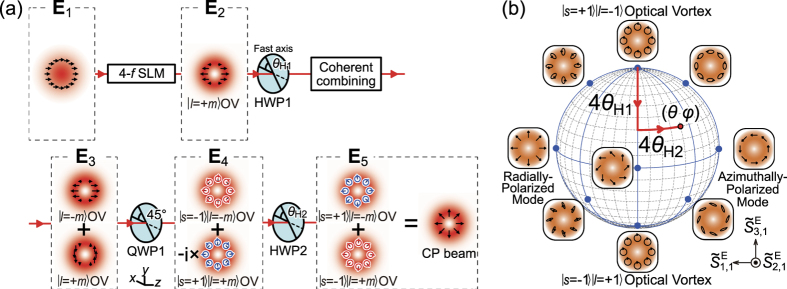
(**a**) An outline drawing of generating arbitrary CP broadband pulses by use of coherent beam combining. SLM, a spatial light modulator; HWP1,2, super-achromatic half-wave plates; QWP1, a super-achromatic quarter-wave plate; In this figure, the case for *m* = 1, *θ*_H1_ = *π*/8( = 22.5°), *θ*_H2_ = −*π*/8( = −22.5°) is drawn. (**b**) The relationship of the rotational angles *θ*_H1_ and *θ*_H2_ to a generated beam state (*m* = 1).

We here describe the basic concept of generating arbitrary CP broadband pulses (Fig. 1(a)). The detail of the experimental setup is shown in Supplementary Fig. S1. First, *x*-polarized 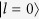 broadband (or ultrashort) pulses are converted into *x*-polarized 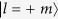 optical vortex (OV) by the spatial light modulator in the 4-*f* configuration (4-*f* SLM). Here, *l* is referred to as the azimuthal index of LG modes^41^. A super-achromatic half-wave plate (HWP1) based on the design by Pancharatnam^42^ and a coherent combining system coherently superpose *x*-polarized 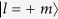 and *y*-polarized 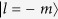 OV broadband pulses, whose energy ratio is controlled by HWP1; 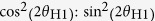. After that, the *x*- and *y*-polarized components of ***E***_3_ are converted into 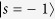 and 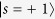 circularly polarized states by a super-achromatic quarter-wave plate (QWP1), respectively. Here, *s* is the spin angular momentum of photon in units of *ħ*^38^. The pulse passes through a super-achromatic half-wave plate (HWP2), following which the sign of spin angular momentum of light is flipped^36^ and the relative phase between 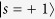 and 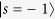 states can be adjusted by the rotation angle of HWP2 *θ*_H2_:


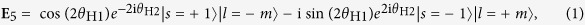


which gives *m*th CP broadband pulses^37^. The normalized extended Stokes parameters (see Supplementary materials for definition) of the pulse state is


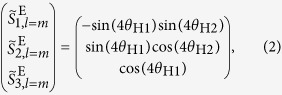


which is represented by the point (*θ*, *ϕ*) = (4*θ*_H1_, *π*/2 + 4*θ*_H2_) on the extended Poincaré sphere (Fig. 1(b)). Hence arbitrary manipulation of CP broadband pulse state can be achieved by adjusting the rotation angles of HWP1 and HWP2. In the present paper, we characterize generated pulse states and spatial symmetry by using parameters of the normalized extended Stokes vectors and the *l*th DOP-SD 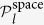, respectively. The definition of 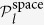 is in Supplementary materials.

### Full quantitative analysis of cylindrically polarized states

**Figure 2:**
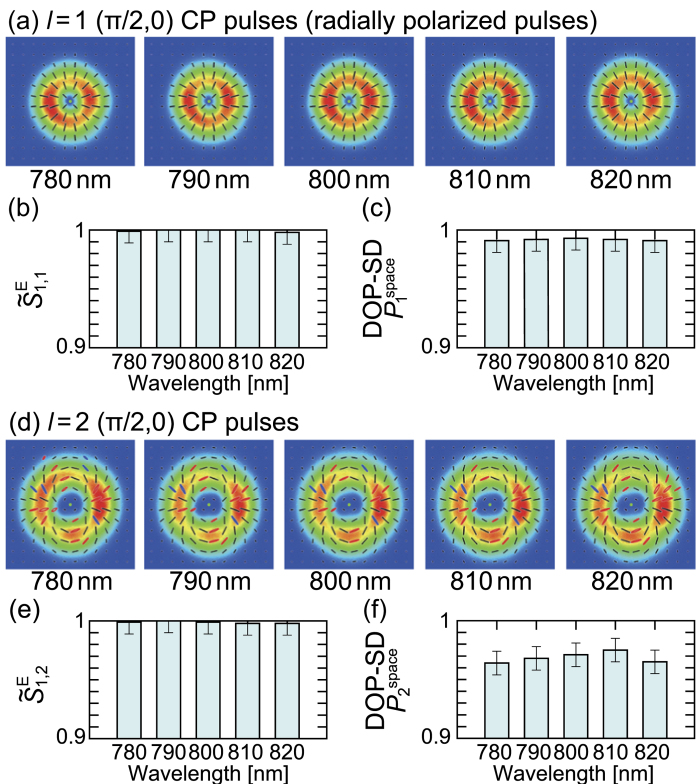
Characterization results of *l* = 1 (**a–c**) and *l* = 2 (**d–f**) (*π*/2, 0) CP broadband pulses. (**a**,**d**) spectrally-resolved polarization distribution of generated *l* = 1 and *l* = 2 CP pulses, respectively. These polarization distributions are colored under the following rule: red, left-handed elliptical polarization; blue, right-handed elliptical polarization; black, linear polarization. The green points at the center of images represent the origins (*x*, *y*) = (0, 0). (**b**,**c**) characterization results of 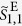 and 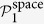 for *l* = 1 (*π*/2, 0) CP pulses (or radially polarized pulses), respectively. (**e**,**f**) characterization results of 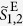 and 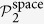 for *l* = 2 (*π*/2, 0) CP pulses, respectively.

We respectively generated seven states for *l* = 1 and *l* = 2 CP broadband pulses: (*θ*, *ϕ*) = (0, 0), (*π*/4, 0), (*π*/4, *π*/4), (*π*/4, *π*/2), (*π*/2, 0), (*π*/2, *π*/4), (*π*/2, *π*/2). For simplicity, (*θ*, *ϕ*) is omitted hereafter. The light source used is a Ti:sapphire laser amplifier (center wavelength 800 nm, bandwidth of ~40 nm, pulse duration ~25 fs, and repetition rate 1 kHz). Figure 2 shows characterization results for *l* = 1 (*π*/2, 0) and *l* = 2 (*π*/2, 0) CP pulses as typical examples. Spectrally-resolved polarization distributions are shown in Fig. 2(a,d); (a) is for *l* = 1 (*π*/2, 0) CP pulses (or radially polarized pulses) and (d) is for *l* = 2 (*π*/2, 0) CP pulses. From the polarization distributions in Fig. 2(a,d), the values of 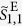 (Fig. 2(b)) and 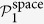 (Fig. 2(c)), and 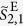 (Fig. 2(e)) and 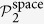 (Fig. 2(f)) in individual spectral ranges were computed.

**Figure 3:**
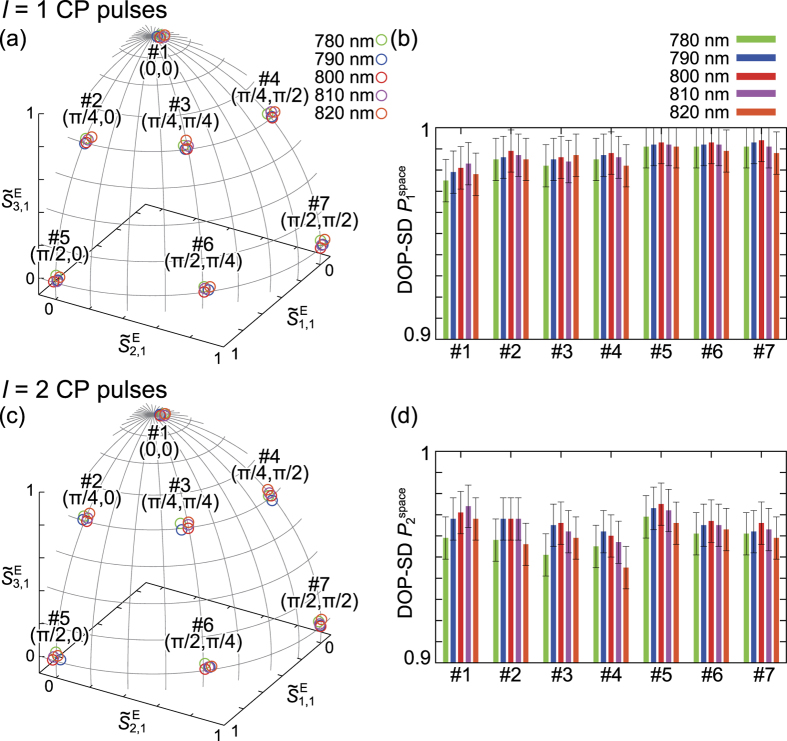
Spectrally-resolved characterization results for *l* = 1 (**a,b**) and *l* = 2 (**c,d**) CP pulses. The seven CP pulse states are realized in every azimuthal index *l*. (**a**,**c**) The values of normalized ESPs 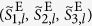 for *l* = 1 and *l* = 2 CP pulse states plotted on the extended Poincaré sphere, respectively. (**b**,**d**) DOP-SD 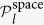 of *l* = 1 and *l* = 2 CP pulse states corresponding to (**a**,**c**), respectively.

The characterization results for all states are described in Fig. 3; (a) and (b) are for *l* = 1 CP pulse states and (c) and (d) are for *l* = 2 CP pulse states. Figure 3(a,c) respectively represent the *l* = 1 and *l* = 2 extended Poincaré sphere, on which the spectrally-resolved values of normalized ESPs 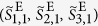 and 
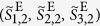
 in *l* = 1 and *l* = 2 CP states are plotted. The spectrally-resolved values of DOP-SD corresponding to the CP states in Fig. 3(a,c) are shown in Fig. 3(b,d), respectively.

All polarization distributions of *l* = 1 (*π*/2, 0) CP pulses at measured wavelengths (780, 790, 800, 810 and 820 nm) in Fig. 2(a) are almost purely radially polarized. This fact is well indicated by the obtained results that 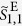 and 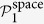 were respectively over 0.99 and 0.98 in all spectral regions (Fig. 2(b,c)). Since 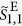 is associated with the energy ratio between (*π*/2, 0) (radially polarized) state and (*π*/2, *π*) (azimuthally polarized) state^38^, which is given by 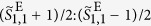, over 99% energy of the temporally- and spatially-perfect-polarized^37^ (TSPP) state was radially polarized. Moreover, DOP-SD 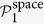 enables us to evaluate the over 98% of the temporally-perfect-polarized state of the generated pulses were TSPP state. Consequently, the pulses generated from a coherent combining system had high purity of *l* = 1 (*π*/2, 0) CP state and high symmetry of polarization distribution.

From Fig. 2(e,f), *l* = 2 (*π*/2, 0) CP pulses similarly had high purity (over 99% in energy ratio) of *l* = 2 (*π*/2, 0) CP state and high symmetry (around 97% in energy ratio) in *l* = 2 CP state, though *l* = 2 pulses were slightly inferior to *l* = 1 pulses with regard to symmetry. Contamination of elliptical polarization in the polarization distribution (Fig. 2(d)) apparently affects the degradation in DOP-SD compared to that of *l* = 1 (*π*/2, 0) CP pulses.

**Figure 4:**
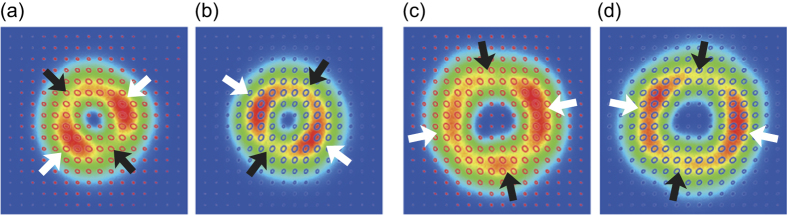
(**a**–**d**) The intensity and polarization distributions of (*l*, *θ*_H1_) = (**a**) (1, 0), (**b**) (1, *π*/4), (**c**) (2, 0) and (**d**) (2, *π*/4) cases. The experimental measurements of (**a**,**c**), and (**b**,**d**) are conducted under blocking the blue blanch and the magenta branch in Supplementary Fig. S1, respectively. The white and black arrows are placed to emphasize the twofold symmetry of the intensity patterns. All the polarization distributions are colored under the rule in Fig. 2.

The contamination comes from two factors. One is the deformation of incident OV pulses into the coherent combining system; the other is the degradation of extinction ratio of the polarizing beam splitter in the coherent combining system because of inclining incident angle. Figure 4(a–d) respectively depict the intensity and polarization distributions of (*l*, *θ*_H1_) = (1, 0), (1, *π*/4), (2, 0) and (2, *π*/4) cases. The measurements of Fig. 4(a,c) and (b,d) are respectively conducted under blocking the blue blanch and the magenta branch in Supplementary Fig. S1, which means **E**_5_ should be proportional to 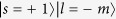 and 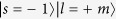. However, these intensity distributions are of twofold symmetry rather than axisymmetry. This result is attributed to the slight superimposition of 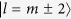 component on 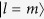 OV pulses because of deformation passing through optic elements. Though the polarization distribution should be circularly polarized, the polarization states are elliptic. The fact can be ascribed to the degradation of extinction ratio of the polarizing beam splitter in the coherent combining system because of inclining incident angle (in other words, the contamination of *s*- and *p*-polarized components at the polarizing beam splitter). The actual electric field of **E**_5_ is approximately described as


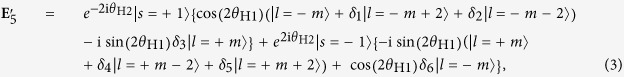


where *δ*_1,2,4,5_ and *δ*_3,6_ are superposition coefficients associated with the deformation and the elliptical polarization, respectively. When *m* = 1, the individual unwanted terms 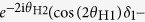 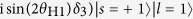 and 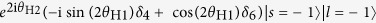 can be partly cancelled. However, in the *m* = 2 case, the unwanted terms are 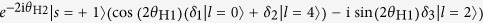 and 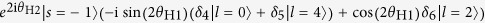, which cannot be cancelled. The contamination of terms except 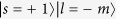 and 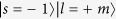 leads to degradation of *C*_|*m*−1|_ rotational symmetry. The value of DOP-SD of *l* = 2 CP pulses are thus smaller than that of *l* = 1 pulses.

Figure 3(a,c) respectively indicate the spectral dependence of polarization states of *l* = 1 and *l* = 2 CP pulses. All the pulse states have quite low spectral dependences thanks to optics for broadband pulses such as super-achromatic wave plates and a low-group-velocity-dispersion polarizing beam splitter. All the values of DOP-SD for *l* = 1 and *l* = 2 CP pulses have low spectral dependence (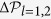 ≲ 0.01), while the DOP-SD values for *l* = 2 CP pulses are somewhat less than those for *l* = 1 CP pulses by 0.02 to 0.03 (Fig. 3(b,d)) because of the previously described reasons. These results clearly show that our system employing coherent beam combining is able to generate arbitrary CP broadband pulse states with high symmetry and low spectral dependence, which is fully-quantitatively investigated by ESPs and DOP-SD with high precision.

### Comparison with simulation

**Figure 5:**
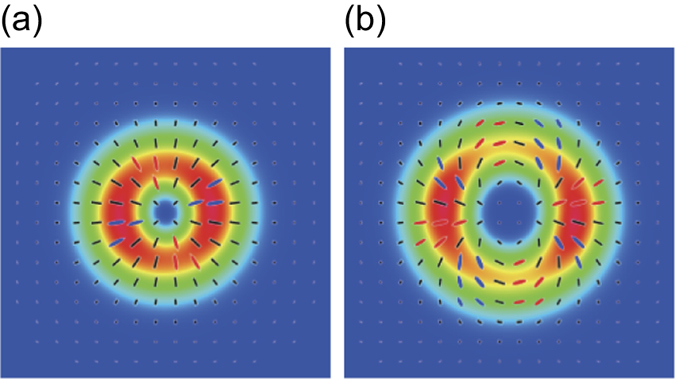
The simulation results for (**a**) *l* = 1 (*π*/2, 0) and (**b**) *l* = 2 (*π*/2, 0) pulse states. All the polarization distributions are colored under the rule in Fig. 2.

In this section, we mention the comparison between the experimental and the simulation results. We conducted simulation for *l* = 1 (*π*/2, 0) and *l* = 2 (*π*/2, 0) CP states. The simulation results are shown in Fig. 5(a,b) and Table 1. Both intensity and polarization distributions in Fig. 5(a,b) well agree with that of the experimental results for *l* = 1 (*π*/2, 0) (Fig. 2(a)) and *l* = 2 (*π*/2, 0) (Fig. 2(d)) states, respectively. The values of 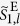 and DOP-SD 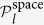 in Table 1 are also in good agreement with the experimental results in Fig. 2(b,e) and (c,f), respectively. In particular, there is a small (~0.02) difference between *l* = 1 and *l* = 2 cases in the simulation results for DOP-SD, which also appears in the experimental results. Therefore, it should be stressed that our measurement method is able to detect such small asymmetricity.

## Perspective

It has been pointed out that precise measurement of polarization state is important in quantum information^43^. Applications using polarized pulses such as material processing^44^, magneto-optical storage^45^ and nonlinear spectroscopic polarimetry^40^ also need to know their polarization states precisely. Using CP pulses instead of the conventional uniform polarized pulses is a manner to extend the degree of freedom in these applications, which have been already demonstrated in quantum information science^5^,^6^,^7^, material processing^16^ and nonlinear spectroscopic polarimetry^40^. Our fully-quantitative measurement method for CP pulses hence can improve the sophistication of these applications.

We think that frequency chirp compensation can be easily achieved because of optics components for broadband pulses in our system. Characterization results in Fig. 3(a,c) show that the dispersions of spectrally-resolved polarization states in individual CP pulse states are small (≲0.05 in propagation distance on the extended Poincaré sphere). CP ultrashort pulses with steady polarization state in the pulse duration, which is especially important for applications for magneto-optical storage and nonlinear spectroscopic polarimetry, can be therefore generated with our system. Our experimental setup, where the accessible spectral range covers the region from 690 nm to 1080 nm (limited by the polarizing beam splitter and half-wave plates), offers us the capability of generating ultrashort CP pulses below 10 fs without polarization distribution dispersion. Moreover, by insertion of a femtosecond polarization pulse shaper^46^ after the 4-*f* SLM system, in place of HWP1 and HWP2, our experimental setup will be able to generate the CP pulses with arbitrary control of temporal CP states on one extended Poincaré sphere. Although the issue of fully-spatiotemporal characterization method for ultrashort pulses with nonuniform polarization distribution still remains, our measurement method is quite useful for precise characterization of ultrashort pulses.

The good agreement between the experimental and simulation results indicates that the degradation in DOP-SD is ascribed to the deformation of incident OV pulses and the contamination of perpendicular polarized components at the polarizing beam splitter, and ensures that we can quantitatively investigate even the small differences of rotational symmetry of polarization distributions or the small contamination of unwanted modes by using DOP-SD. At least 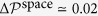 is significant and detectable in our measurement system. Though the earlier studies have not taken account of DOP-SD, DOP-SD as well as ESPs is an important parameter for full-quantitative characterization of CP states.

## Methods

### Generation of broadband optical vortex pulses

The generated pulses from a Ti:sapphire laser amplifier are attenuated by ND filters, following which the 4-*f* SLM converts into *x*-polarized *l* = 1, *p* = 0 or *l* = 2, *p* = 0 OV pulses. Here, *p* denotes the radial index of LG modes^41^. The 4-*f* configuration in the SLM system enables us to compensate for angular dispersion^47^,^48^. We furthermore utilize a complex-amplitude modulation technique with a phase-only SLM^49^,^50^ as means to convert to broadband arbitrary single LG mode OV pulses.

### Finding the zero delay in the coherent combining system

Using a polarizer (POL2 in Supplementary Fig. S1) and a spectrometer, we find the zero delay with the aid of the spectrum interference method. A charge-coupled-devise (CCD1 in Supplementary Fig. S1) monitors the intensity profile of the *x*-polarized component of **E**_4_ in order to ensure the delay time is unchanged within the polarization measurement.

### Measuring polarization distributions

In the polarization measurement system, the pulses are spectrally-resolved by bandpass filters (BPF in Supplementary Fig. S1; center wavelengths, 780, 790, 800, 810, 820 nm; bandwidths, 10 nm), then their polarization distributions are acquired by using a rotating-retarder type imaging polarimeter^51^, which is composed of an achromatic quarter-wave plate (QWP2 in Supplementary Fig. S1), a Glan-Laser polarizer (GLP in Supplementary Fig. S1) and a charge-coupled-devise camera (CCD2 in Supplementary Fig. S1). From the polarization distribution, we computed the normalized extended Stokes vectors 
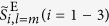
 and the *m*th DOP-SD 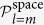. Here, the origins (*x*, *y*) = (0, 0) on the recorded images are set to maximize the *m*th DOP-SD.

### Simulation

We respectively evaluated *δ*_1,2,4,5_ and *δ*_3,6_ from the intensity and the polarization distributions in Fig. 4(a–d) (the values are in Table 1). The intensity distributions were plotted by using the following equation based on equation 3:


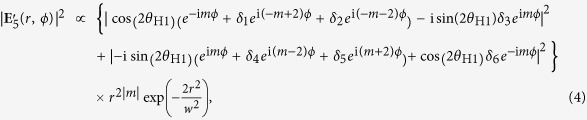


where (*r*, *ϕ*) is the circular polar coordinates and *w* is a parameter for the beam size. We have made simulations under the various conditions of *δ*_1,2,4,5_, and confirmed that the values of 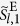 and 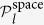 hardly changed.

## Additional Information

**How to cite this article**: Suzuki, M. *et al.* Full Quantitative Analysis of Arbitrary Cylindrically Polarized Pulses by Using Extended Stokes Parameters. *Sci. Rep.* **5**, 17797; doi: 10.1038/srep17797 (2015).

## Supplementary Material

Supplementary Information

## Figures and Tables

**Table 1:** Simulation conditions and values of 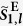 and DOP-SD 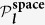.

*l* (= *m*)	*δ*_1_	*δ*_2_	*δ*_3_	*δ*_4_	*δ*_5_	*δ*_6_	*θ*_H1_	*θ*_H2_	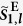	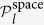
1	−0.08*e*^2.20i^	0	0.14*e*^−2.52i^	0	0.08*e*^−0.94i^	0.15*e*^−0.44i^	*π*/8	−*π*/8	0.999	0.989
2	0.08*e*^0.30i^	0	0.14*e*^−2.52i^	0	0.08*e*^0.34i^	0.15*e*^−0.44i^	*π*/8	−*π*/8	0.999	0.972
