# Supplementary material for: Full Quantitative Analysis of Arbitrary Cylindrically Polarized Pulses by Using Extended Stokes Parameters
Source: Sci Rep. 2015 Dec 10;5:17797. doi: 10.1038/srep17797 (PMC4674746; doi:10.1038/srep17797)
Supplement: Supplementary Information [file srep17797-s1.pdf]

Full Quantitative Analysis of Arbitrary Cylindrically Polarized Pulses by Using Extended Stokes Parameters - Supplementary information

Masato Suzuki, Keisaku Yamane, Kazuhiko Oka, Yasunori Toda, and Ryuji Morita
Department of Applied Physics, Hokkaido University,
*Kita-13, Nishi-8, Kita-ku, Sapporo 060-8628, Japan**

EXPERIMENTAL SETUP

Figure S1 shows an experimental setup. This setup is composed of 5 parts; 4-f SLM, coherent combining, CP beam generation, spectral interference and monitoring, and polarization measurement systems. In this section, we describe the coherent combining system.

After passing through a super achromatic half wave plate (HWP1), the transverse electric field $\mathbf{E}_{\mathrm{HWP1}}$ is represented by

$$\mathbf{E}_{\mathrm{HWP1}}(x,y,t) = E(t)f(x,y)\begin{pmatrix}\cos 2\theta_{\mathrm{H1}}\\ -\sin 2\theta_{\mathrm{H1}}\end{pmatrix}e^{\mathrm{i}m\phi}, \tag{S1}$$

where $E(t)$ is the temporal envelope of generated ultrashort pulses, $f(x,y)$ is an amplitude function without phase ramp around the beam axis, and m is the topological charge of OV (or the azimuthal index of LG modes). The direction of pulses is changed by 90° by a periscope (P1) whose two mirrors are placed with a twist. The periscope swaps the electric components E_x and E_y. The Jones vector thereby is rewritten as following:

$$E(t)f(x,y)\begin{pmatrix}\cos 2\theta_{\mathrm{H1}}\\ -\sin 2\theta_{\mathrm{H1}}\end{pmatrix}e^{\mathrm{i}m\phi} \to E(t)f(-x,-y)\begin{pmatrix}-\sin 2\theta_{\mathrm{H1}}\\ \cos 2\theta_{\mathrm{H1}}\end{pmatrix}e^{\mathrm{i}m\phi}. \tag{S2}$$

The pulse is divided into two by a low-group-velocity-dispersion polarization beam splitter (PBS). The numbers of reflection in the blue branch and the magenta branch in Fig. S1 are 6 and 7, respectively. Thus, the beam cross-sectional profile of the pulses in the magenta branch is flipped ($f(-x,-y)e^{\mathrm{i}m\phi} \to f(x,-y)e^{-\mathrm{i}m\phi}$). An achromatic half-wave plate (HWP2) makes a polarization rotation of pulses in the both blue and magenta branches by 90°. The x-polarized $l=-m$ and y-polarized $l=m$ OV are hence coherently combined at PBS:

$$\mathbf{E}_3(x,y,t) = f(-x,-y)\begin{pmatrix}E(t+\tau)\cos 2\theta_{\mathrm{H1}}e^{-\mathrm{i}m\phi}\\ E(t)\sin 2\theta_{\mathrm{H1}}e^{\mathrm{i}m\phi}\end{pmatrix}. \tag{S3}$$

Here τ is delay time between pulses in the blue and magenta branches, which is controlled by the piezo driver to be $\tau = 0$. We assume that the amplitude profile f has a bilaterally symmetric $f(x,-y) = f(-x,-y)$ since OV has doughnut-shaped intensity profile.

DECOMPOSITION OF A EXTENDED STOKES VECTOR AND DEFINITION OF THE NORMALIZED EXTENDED STOKES VECTOR

With $\mathcal{P}_l^{\mathrm{space}}$ and $S_{0,l}^{\mathrm{E,(P)}}$ (the integral of the amount proportional to the time-averaged intensity of light with temporally perfect polarization state [S1]), the lth extended Stokes vector can be split into three parts

$$\begin{pmatrix}S_{0,l}^{\mathrm{E}}\\ S_{1,l}^{\mathrm{E}}\\ S_{2,l}^{\mathrm{E}}\\ S_{3,l}^{\mathrm{E}}\end{pmatrix} = \begin{pmatrix}S_{0,l}^{\mathrm{E}} - S_{0,l}^{\mathrm{E,(P)}}\\ 0\\ 0\\ 0\end{pmatrix} + \begin{pmatrix}S_{0,l}^{\mathrm{E,(P)}}(1-\mathcal{P}_l^{\mathrm{space}})\\ 0\\ 0\\ 0\end{pmatrix} + \begin{pmatrix}S_{0,l}^{\mathrm{E,(P)}}\mathcal{P}_l^{\mathrm{space}}\\ S_{1,l}^{\mathrm{E}}\\ S_{2,l}^{\mathrm{E}}\\ S_{3,l}^{\mathrm{E}}\end{pmatrix}. \tag{S4}$$

---

* E-mail me at: morita@eng.hokudai.ac.jp

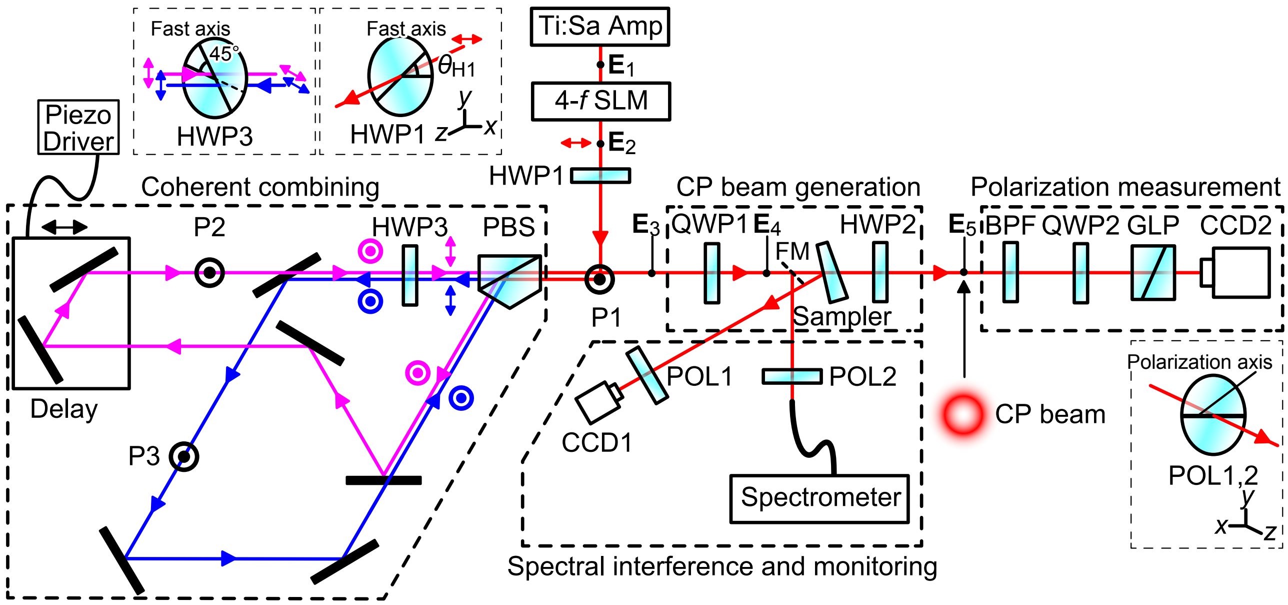

FIG. S1. Setup for generating arbitrary CP broadband pulses by use of coherent beam combining. SLM, a spatial light modulator (available wavelength ranges from 620 nm to 1100 nm); HWP1,2, super achromatic half-wave plates (from 600 nm to 2700 nm); HWP3, an achromatic half-wave plate (from 690 nm to 1200 nm); PBS, a low-group-velocity-dispersion polarizing beam splitter (from 680 nm to 1080 nm); P1-3, periscopes; QWP1, a super achromatic quarter-wave plate (from 600 nm to 2700 nm); QWP2, an achromatic quarter-wave plate (from 690 nm to 1200 nm); FM, a flip mirror; POL1,2, polarizers; Sampler, a beam sampler; BPF, a bandpass filter; GLP, a Glan-Laser polarizer; CCD1,2, charge-coupled-devise cameras.

Here, $\mathcal{P}_l^{\mathrm{space}}$ and $S_{0,l}^{\mathrm{E,(P)}}$ are defined by the following equations:

$$\mathcal{P}_l^{\mathrm{space}} = \sqrt{(S_{1,l}^{\mathrm{E}})^2 + (S_{2,l}^{\mathrm{E}})^2 + (S_{3,l}^{\mathrm{E}})^2} \Big/ \iint_A \sqrt{(S_1)^2 + (S_2)^2 + (S_3)^2}\mathrm{d}x\mathrm{d}y, \tag{S5}$$

$$S_{0,l}^{\mathrm{E,(P)}} = \iint_A \sqrt{(S_1)^2 + (S_2)^2 + (S_3)^2}\mathrm{d}x\mathrm{d}y, \tag{S6}$$

where $S_i = S_i(x, y, z)$ $(i = 1 - 3)$ are the conventional Stokes parameters at the point (x, y, z). The first, second and third terms of equation (S4) are temporally unpolarized, temporally-perfect-polarized but spatially unpolarized (TPPSU), and temporally- and spatially-perfect-polarized (TSPP) Stokes vectors, respectively. Representing the energy ratio between TPPSU and TSPP states in the temporally-perfect-polarized state, lth DOP-SD $\mathcal{P}_l^{\mathrm{space}}$ is thus a measure of symmetry in the lth CP state.

Here, we introduce the normalized ESPs

$$\tilde{S}_{i,l}^{\mathrm{E}} = \frac{S_{i,l}^{\mathrm{E}}}{S_{0,l}^{\mathrm{E,(P)}}\mathcal{P}_l^{\mathrm{space}}} = \frac{S_{i,l}^{\mathrm{E}}}{\sqrt{(S_{1,l}^{\mathrm{E}})^2 + (S_{2,l}^{\mathrm{E}})^2 + (S_{3,l}^{\mathrm{E}})^2}}, \tag{S7}$$

therefore the normalized extended Stokes vector or the normalized TSPP Stokes vector $\tilde{\mathbf{S}}_{\mathrm{TSPP}}^l$ is

$$\tilde{\mathbf{S}}_{\mathrm{TSPP}}^l = \begin{pmatrix} 1 \\ \tilde{S}_{1,l}^{\mathrm{E}} \\ \tilde{S}_{2,l}^{\mathrm{E}} \\ \tilde{S}_{3,l}^{\mathrm{E}} \end{pmatrix}. \tag{S8}$$

From Eq. (S8), the normalized TSPP Stokes vector and its Poincaré sphere respectively correspond to the higher-order

[S2, S3] and the hybrid [S4] Stokes vector and their Poincaré sphere.

[S1] Suzuki, M., Yamane, K., Oka, K., Toda, Y. & Morita, R. Extended stokes parameters for cylindrically polarized beams. *Optical Review* **22**, 179–183 (2015).
[S2] Milione, G., Sztul, H. I., Nolan, D. A. & Alfano, R. R. Higher-order poincaré sphere, stokes parameters, and the angular momentum of light. *Phys. Rev. Lett.* **107**, 053601 (2011).
[S3] Milione, G., Evans, S., Nolan, D. A. & Alfano, R. R. Higher order pancharatnam-berry phase and the angular momentum of light. *Phys. Rev. Lett.* **108**, 190401 (2012).
[S4] Holleczek, A., Aiello, A., Gabriel, C., Marquardt, C. & Leuchs, G. Classical and quantum properties of cylindrically polarized states of light. *Opt. Express* **19**, 9714–9736 (2011).
